# Supplementary material for: Socioeconomic Status and Distance to Reference Centers for Complex Cancer Diseases: A Source of Health Inequalities? A Population Cohort Study Based on Catalonia (Spain)
Source: Int J Environ Res Public Health. 2022 Jul 20;19(14):8814. doi: 10.3390/ijerph19148814 (PMC9322195; doi:10.3390/ijerph19148814)
Supplement: Supplementary file 1 [file ijerph-19-08814-s001.zip › Supplementary Material Table S1.pdf]

Supplementary Material Table S1. Baseline characteristics of cohort by type of cancer and by sex

		Pancreas					Rectum				
		Men		Women			Men		Women		
		n	%	n	%	p	n	%	n	%	p
Age, years	Median (IQR)	68.0 (14.0)		71.0 (16.0)		0.001*[1]	69.2 (16.3)		71.3 (17.6)		0.128 [1]
Age,categories	< 60 years	94	24,8	47	17,6	0.002*	205	21,6	106	20,9	0,226
	60 - 69 years	128	33,8	78	29,2		286	30,2	137	27,0	
	70 - 79 years	128	33,8	99	37,1		299	31,6	160	31,5	
	>= 80 years	29	7,7	43	16,1		157	16,6	105	20,7	
ASA	ASA I	6	1,6	11	4,1	0,300	57	6,0	32	6,3	0.043*
	ASA II	99	26,1	69	25,8		462	48,8	276	54,3	
	ASA III	99	26,1	63	23,6		338	35,7	148	29,1	
	ASA IV	9	2,4	4	1,5		36	3,8	13	2,6	
	Missing	166	43,8	120	44,9		54	5,7	39	7,7	
TNM staging	0/I	40	10,6	23	8,6	0,901	123	13,0	78	15,4	0,145
	II	151	39,8	105	39,3		234	24,7	100	19,7	
	III	13	3,4	11	4,1		490	51,7	271	53,3	
	IV	6	1,6	3	1,1		88	9,3	48	9,4	
	non-stageable	67	17,7	54	20,2		12	1,3	11	2,2	
	Missing	102	26,9	71	26,6						
Income	High and medium	125	33,0	40	15,0	<0.001*	283	29,9	96	18,9	<0.001*
	Low	245	64,6	217	81,3		621	65,6	385	75,8	
	Very low	9	2,4	10	3,7		43	4,5	27	5,3	
Distance, Km	Median (IQR)<br>min - max	7,76 (33,21)<br>0,53 - 246,07		5,94 (32,01)<br>0,63 - 191,10		0.635 [1]	4,43 (8,51)<br>0,39 - 134,86		4,28 (9,52)<br>0,19 - 64,04		0.460 [1]
Distance, cat	0 - 10 km	221	58,3	159	59,6	0,952	676	71,4	364	71,7	0,587
	>10 km - <P90	120	31,7	82	30,7		183	19,3	90	17,7	

	>= P90	38	10,0	26	9,7		88	9,3	54	10,6	
Intermediate centre[2]	Intermediate centre	165	43,5	108	40,4	0,434	112	11,8	71	14,0	0,239
	Reference centre RC	214	56,5	159	59,6		835	88,2	437	86,0	
Total patients		379	58,7	267	41,3		947	65,1	508	34,9	

Distance: km between the residential location of patients and centre of surgery
P90 distance: ancreatic cancer=89,14Km, Rectal cancer=29,70Km.

1. U Mann-Whitney test for median comparison

2. Intermediate centre (The proximity centre close to residential location is not the RC for sugery) / 1. RC (The proximity centre is the RC for surgery)

IQR: Interquartile range

Missing: no data found

P values below 0.05 (two-sided) were considered to indicate statistical significance.

* Chi-square test
