# Supplementary material for: Socioeconomic Status and Distance to Reference Centers for Complex Cancer Diseases: A Source of Health Inequalities? A Population Cohort Study Based on Catalonia (Spain)
Source: Int J Environ Res Public Health. 2022 Jul 20;19(14):8814. doi: 10.3390/ijerph19148814 (PMC9322195; doi:10.3390/ijerph19148814)
Supplement: Supplementary file 1 [file ijerph-19-08814-s001.zip › Supplementary Material Table S2.pdf]

Supplementary Material Table S2. Description of distance by age, stage, income and pathology

		Pancreas			Rectum		
		Median	(IQR)	p1	Median	(IQR)	p1
Age,categories				0,383			0.019[*,1]
	< 60 years	6,68	32,51		5,63	9,85	
	60 - 69 years	8,59	40,06		4,45	8,48	
	70 - 79 years	7,03	31,17		4,21	9,07	
	>= 80 years	4,88	19,86		3,75	7,66	
Stage				0,209			0,118
	0/I	5,24	9,02		3,76	7,63	
	II	7,95	32,99		4,18	7,99	
	III	5,07	17,98		4,47	9,27	
	IV	5,54	3,17		5,58	15,81	
	non-stageable	7,63	34,24		4,15	5,23	
	Missing	7,76	40,30				
Income				0.022*[,2]			0.038*[,3]
	High and medium	5,39	18,14		3,94	7,44	
	Low	8,26	35,68		4,52	10,18	
	Very low	6,42	9,77		3,99	7,47	

P90 distance: ancreatic cancer=89,14Km, Rectal cancer=29,70Km.

p1. Kruskal–Wallis H test

1. U Mann-Whitney test for median comparison: statistical significant difference between categories >80 and <60 (p=0.015)

2. U Mann-Whitney test for median comparison: statistical significant difference between "High and Medium" and "Low" income categories (p=0.024)

3. U Mann-Whitney test for median comparison: statistical significant difference between "High and Medium" and "Low" income categories (p=0.032)

IQR: Interquartile range

Missing: no data found
